# Increased expression of IDO associates with poor postoperative clinical outcome of patients with gastric adenocarcinoma

**DOI:** 10.1038/srep21319

**Published:** 2016-02-18

**Authors:** Hao Liu, Zhenbin Shen, Zhenglin Wang, Xuefei Wang, Heng Zhang, Jing Qin, Xinyu Qin, Jiejie Xu, Yihong Sun

**Affiliations:** 1 Department of General Surgery, Zhongshan Hospital, Fudan University, Shanghai 200032, China; 2 Department of Biochemistry and Molecular Biology, School of Basic Medical Sciences, Fudan University, Shanghai 200032, China

## Abstract

Clinical significance of 2,3-dioxygenase (IDO) has been studied in types of tumors, but the role that IDO played in gastric adenocarcinoma (GAC) is still unclear. Here, we aim to investigate the prognostic value of IDO expression in patients with GAC. We examined intratumoral IDO expression in retrospectively enrolled 357 patients with GAC undergoing gastrectomy at Zhongshan Hospital of Fudan University in 2008 by immunohistochemical staining. The Kaplan-Meier method and Cox regression models were used to evaluate the prognostic value of IDO expression and its association with clinical pathological factors. We generated a predictive nomogram by integrating IDO expression with the TNM staging system for overall survival of GAC patients. High expression of intratumoral IDO predicted a dismal outcome. Intratumoral IDO expression gave a further discrimination for the prognosis of GAC patients. By Cox multivariate analysis, IDO expression was defined as an independent prognosticator. The generated nomogram performed well in predicting the 3- and 5-year overall survival of GAC patients. Conclusively, IDO is a potential prognostic biomarker for overall survival of patients with GAC after gastrectomy.

Despite the steadily decreasing incidence and mortality rates in the majority of the world, gastric adenocarcinoma (GAC) remains the fourth most common malignancy and the third leading cause of cancer death worldwide. Moreover, incidence rates are much higher in East Asia, particular in Korea, Japan and China, than that in North America and most parts of Africa^1^. Due to the mild and atypical symptoms at the early stage, over 80% of the patients were clinically diagnosed at an advanced stage, which generally indicated a dismal outcome^2^,^3^.

Currently, UICC/AJCC TNM system is the widely used risk stratification system for patients with GAC. The underlying molecular and cellular processes during the carcinogenesis of GAC are ignored in this system while broad evidences emerge to state that the disease is heterogeneous with unpredictable nature history^4^. Thus, illumination of the involved molecules and the underlying mechanisms in the development and progression of GAC might contribute to disease prognosis and provide novel therapeutic targets for the treatment.

Indoleamine 2,3-dioxygenase (IDO) is responsible for the first enzymatic step of tryptophan catabolism by the kynurenine pathway performed as the rate-limiting enzyme^5^. Increasing evidences implicated that IDO, as well as tryptophan catabolism, generated immune tolerance to foreign antigens in tissue microenvironment. Within a tryptophan-depleted microenvironment, the expression of IDO shields the tumor from an immune response against it either by a direct suppression of T cell function and proliferation or indirect suppression of anti-tumor immune responses by accumulation of IDO catabolites into the environment^6^. Moreover, IDO expression was also reported to be associated with M2-polarization of tumor-associated macrophages and impaired function of nature killer cells^7^,^8^,^9^.

In non-small cell lung cancer^10^, colorectal cancer^11^,^12^, ovarian cancer^13^, endometrial cancer^14^, breast cancer^15^, as well as in childhood acute myeloid leukemia^16^, aberrant expression of IDO had been determined as a prognosticator for poor prognosis. These suggested a therapeutic potential for IDO inhibitors. 1-methyl tryptophan (1MT) is a specific inhibitor of IDO^17^. Several studies have offered evidence that IDO inhibition with 1MT could exert antitumor effects^18^. In H. pylori-infected human gastric mucosa, an enhanced expression of IDO was detected^19^. However, divergent results have been reported about IDO expression in GAC and its correlation with tumor stage^20^,^21^. The expression of IDO in GAC and its clinical significance have not been fully clarified and need further study.

In this study, we sought to investigate IDO expression in patients with GAC and explore its correlation with the clinical pathological characteristics and clinical outcome. Moreover, a nomogram integrated IDO expression and depth of tumor invasion, lymph node metastasis status was constructed to present a quantitative assessment for the 3- and 5-year overall survival in GAC patients after surgery.

## Results

### IDO expression in gastric adenocarcinoma

**Figure 1:**
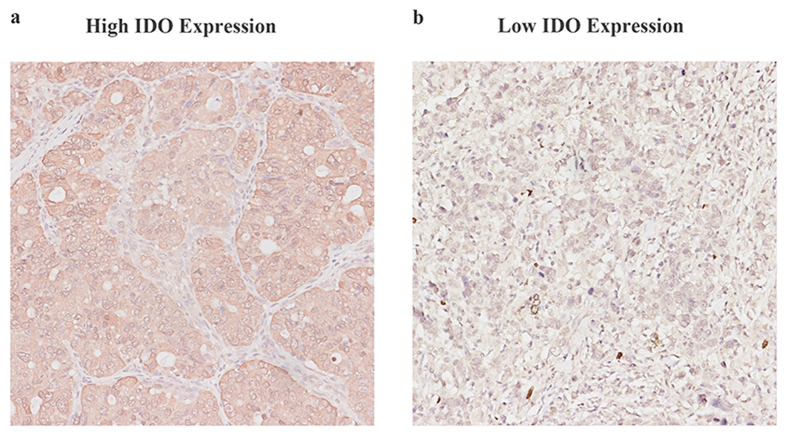
Representative images for IDO expression in GAC. Gastric adenocarcinoma with high IDO expression (**a**) and low IDO expression (**b**). Magnification 200×.

The positive staining of IDO was observed in the cytoplasm and/or on the membrane of neoplastic epithelial cells and mononuclear cells in the stroma (Fig. 1a,b). The integrated optical density (IOD) of the immunostaining in each specimen varied greatly in the cancer tissue. The measured IOD of the staining in tumor tissue was 352.25 ± 291.58 (median, 284.00; range from 1.62 to 587.78). With the X-tile software, the cut-off point was 282, which was determined using the method of minimum p value. Thus, 175 patients (49%) were separated into the IDO low expression subgroup and 182 patients (51%) were separated into the IDO high expression subgroup.

### Correlations between IDO Expression and clinical pathological features

The relationship between clinical pathological characteristics and IDO expression is shown in Table 1. Intratumoral IDO expression was significantly associated with the depth of tumor invasion (*p* = 0.045) and lymph node metastasis (*p* < 0.001).For the other clinical pathological characteristics, no significant association was found.

### Prognostic evaluation of IDO expression in gastric adenocarcinoma

**Figure 2:**
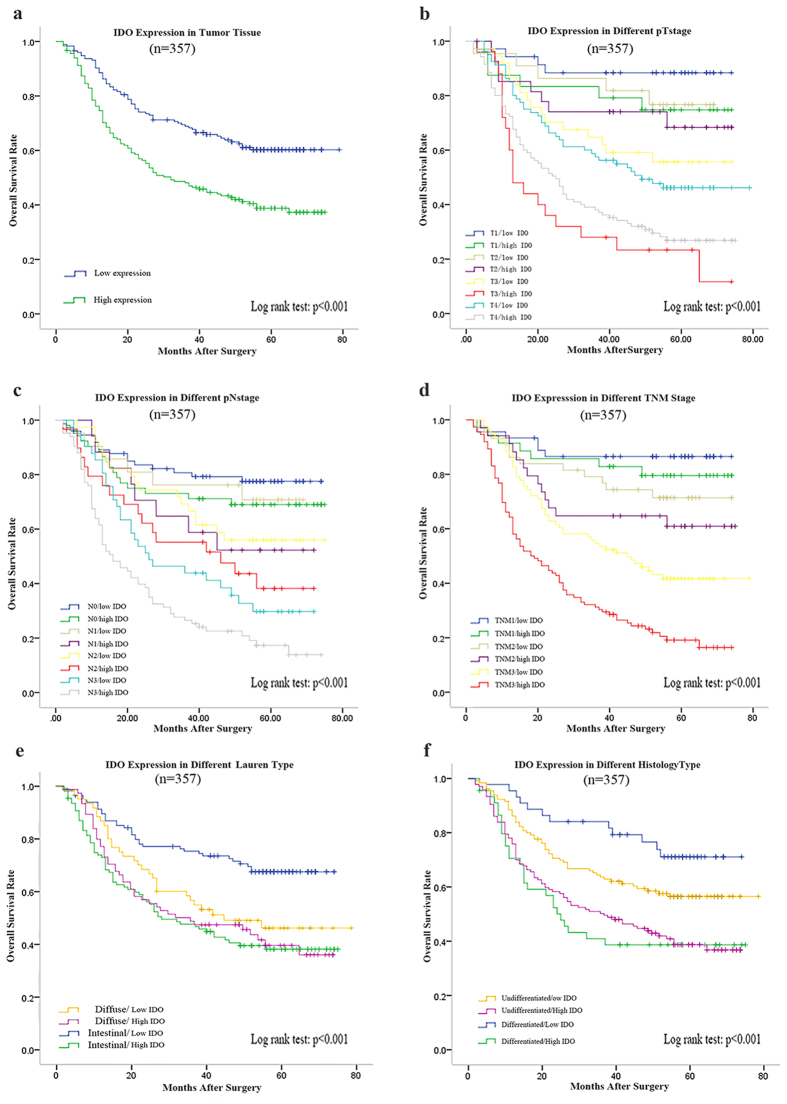
Kaplan-Meier analysis for overall survival of patients with GAC according to IDO expression. (**a**) Kaplan-Meier analysis for overall survival according to intratumoral IDO expression. Kaplan-Meier analysis for overall survival according to intratumoral IDO expression in patients with different tumor invasion depth (**b**); different lymph node metastasis status (**c**); different pTNM stage (**d**); different Lauren’s classification (**e**); different histological type (**f**). *p* value, calculated by Log rank test, <0.05 was regarded as statistically significant.

By separating the patients into two subgroup as mentioned above, we applied Kaplan-Meier analysis to determine the overall survival. Log rank test was used to give the *p* values. As shown in Fig. 2a, high expression of IDO was associated with poor overall survival (*p* < 0.001). The median survival time for IDO low expression group was 51.4 months while that for IDO high expression group was 30.7 months.

**Figure 3:**
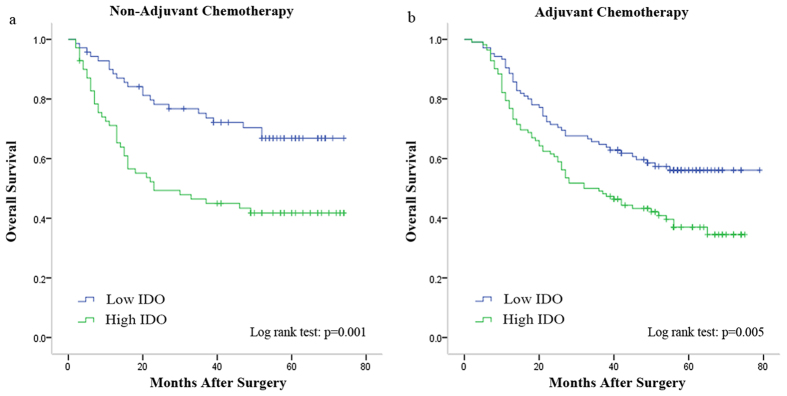
Kaplan-Meier analysis for overall survival in GAC patients with or without adjuvant chemotherapy according to intratumoral IDO expression. Kaplan-Meier analysis for overall survival according to intratumoral IDO expression in patients with adjuvant chemotherapy (**a**) and without adjuvant chemotherapy (**b**).

The overall survival of the two subgroups was significantly different in different tumor invasion depth (Fig. 2b), lymph node metastasis status (Fig. 2c), TNM stage (Fig. 2d), Lauren’ classification (Fig. 2e) and histology (Fig. 2f), with all the *p* values less than 0.001. Furthermore, statistically significant difference in overall survival was found between the two subgroups in patients without adjuvant chemotherapy (*p* = 0.001, Fig. 3a), as well as in patients with adjuvant chemotherapy (*p* = 0.005, Fig. 3b). However, in Fig. 2d, for example, the overall survival for the patients of TNM I stage did not differ significantly in the IDO low and high expression subgroups although the *p* value for the whole model was less than 0.001.

Thus, we calculated the hazard ratios of IDO high expression in GAC in different tumor invasion depth, lymph node metastasis status, TNM stage, Lauren’ classification, histology and adjuvant chemotherapy status respectively to give a further analysis. As shown in Table 2, we found that in T3 and T4, N3, TNM III, Lauren’s intestinal type, patients of IDO high expression group had a significantly increased risk. Meanwhile, patients in IDO high expression group suffered a higher risk of death regardless of adjuvant chemotherapy and histological type.

**Figure 4:**
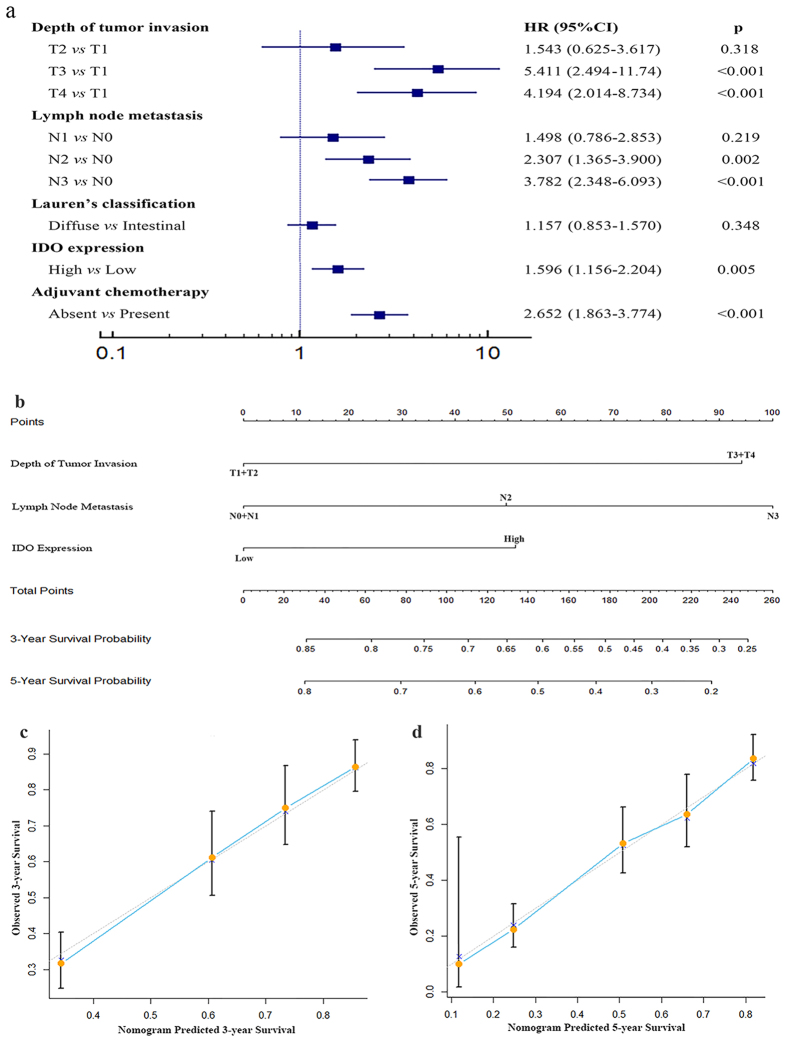
Cox multivariate analysis for independent prognostic factors and nomogram for predicting 3- and 5-year overall survival in patients with GAC. (**a**) Cox multivariate analysis identified the independent prognostic factors for overall survival for patients with GAC. (**b**) Nomogram for predicting clinical outcomes integrated IDO expression (High/Low) with tumor depth (T1 + 2/T3 + 4) and lymph nodes metastasis (N0 + N1/N2/N3). In the nomogram, higher total point predicts worse prognosis. Addition of tumor invasion depth (0 for T1 + T2, 94 for T3 + T4), lymph node metastasis (0 for N0 + N1, 51 for N2, 100 for N3), and IDO expression (0 for Low, and 52 for High) for each patients correspondingly gives the total point. (**c**) Calibration plot for nomogram predicted and observed 3-year survival rate. (**d**) Calibration plot for nomogram predicted and observed 5-year survival rate. Calibration curves for nomogram predicted 3- and 5-year overall survival performed well with the ideal model. *Line of dashes*: ideal model; *vertical bars*, 95% confident interval.

In the univariate Cox regression analysis of overall survival, IDO expression was defined as a prognostic factor (*p* < 0.001). Multivariate analysis revealed that intratumoral IDO was a risk factor for death, independent of depth of tumor invasion, lymph node metastasis, Lauren’s classification and adjuvant chemotherapy (Fig. 4a).

### Predictive Nomogram for overall survival of patients with gastric adenocarcinoma

A predictive nomogram was constructed to give a quantitative model to stratify GAC patients into different risks. Independent factors for overall survival selected by multivariate analysis, excluding adjuvant chemotherapy, were integrated in the nomogram. Since T1 and T2, T3 and T4, N0 and N1 had a similar hazard ratio for overall survival (Fig. 4a), we combined T1 with T2, T3 with T4, N0 with N1 in the nomogram. In the nomogram, a higher total point predicts a worse prognosis. The total point was raised by adding the score of tumor depth (0 for T1 + T2, 94 for T3 + T4), lymph node metastasis (0 for N0 + N1, 51 for N2, 100 for N3), and IDO expression (0 for low, and 52 for high) for each patients correspondingly (Fig. 4b). Calibration curves for nomogram predicted 3- and 5-year overall survival were built to give the internal validation, which performed well with the ideal model (Fig. 4c,d). The Harrell’s c-index for the generated nomogram was 0.702 (95% *CI*, 0.663-0.741), higher than that for TNM stage (0.684; 95% *CI*, 0.646–0.722), indicating that the nomogram gave a better prediction for overall survival of the patients.

## Discussion

Studies on IDO expression in various tumors and antigen presenting cells have confirmed that IDO played an immunosuppressive role in carcinogenesis and served as a biomarker for poor prognosis in some tumors^10^,^11^,^12^,^13^,^14^,^15^. However, few studies focused on the expression of IDO and its clinical significance in GAC. Here, we demonstrated the prognostic value of IDO expression in GAC and gave a simple risk classification for the patients according to IDO expression. Cox multivariate analysis revealed IDO expression as an independent prognostic factor for overall survival of the patients. The nomogram model based on the integration of depth of tumor invasion, lymph node metastasis and IDO expression seemed to perform better than TNM staging system in predicting overall survival of the patients.

The immune system gains increasing interests for its crucial role in carcinogenesis of a bunch of tumors, not only for its suppression of tumor growth by ruining cancer cells or inhibiting their invasion, but also for the promotion of tumor progression either by selecting tumor cells that are more accommodative to survive in an immunocompetent host or by establishing requisites in tumor microenvironment that facilitate tumor invasion^22^. Lymphocytes, especially T cells, are very sensitive to particular amino acids in the local environment^23^. Both tryptophan depletion mediated by IDO in a cellular microenvironment and tryptophan catabolites contributed to establish a regulatory environment affecting CD8+ as well as CD4+ T cell function, and not only was tryptophan catabolism an effector pathway of immunologic tolerance, but it also led to GCN2-dependent formation and activity of Treg cells^24^ and myeloid derived suppressor cells (MDSCs)^25^. Further, some studies stated that aberrant expression of IDO was implicated in the polarization of IL-10-secreting M2 immunosuppressive macrophages (CD14+/CD206+)^26^. Thus, it is conceivable that aberrant IDO expression in GAC created an immunosuppressive microenvironment that favored the infiltration and invasion of the primary tumor, which could give a possible explanation to the finding in the study that IDO expression in GAC was associated with depth of tumor invasion and lymph node metastasis (Table 1).

Notably, since Lauren introduced in 1965 GAC occurred in two subtypes, intestinal type and diffuse type, debates have continued whether Lauren’s classification could give a risk stratification in patients with GAC. Our study found that while there was no difference between overall survival rates of IDO high and low expression patients in Lauren’s diffuse type, the overall survival rates significantly differed in Lauren’s intestinal type. That raised the hypothesis that tumors of the two subtypes could totally be two different kind of diseases, although they both occurred in the stomach and were so called “gastric adenocarcinoma”.

In this study, we demonstrated the prognostic value of IDO expression in GAC. According to intratumoral IDO expression, patients could be separated into two subgroups regardless of adjuvant chemotherapy. IDO expression proved to be an independent adverse prognosticator for overall survival in patients with GAC. Stratification analyses revealed IDO expression could give some additional prognostic information in tumors of different stages and of different histology types. Further, we constructed a nomogram by integrating IDO expression, depth of tumor invasion and lymph node metastasis status to give a prediction for the 3- and 5-year overall survival of the patients. Calibration plots and c-indices indicated the generated nomogram performed better than TNM staging system in discriminating the patients with different clinical outcomes.

However, owing to the limitation of retrospective study, we only found a close relationship between IDO expression and tumor stage. The causal relationship between higher IDO expression and progression of GAC remains elusive. The exact role of IDO in the progression of GAC would be investigated in our future work. Moreover, the number of patients enrolled in the study is relatively small. A large, multi-center, prospective data is needed to validate these results.

In conclusion, we have identified aberrant expression of IDO in GAC as an independent prognosticator, which could be incorporated with depth of tumor invasion and lymph node metastasis status to generate a nomogram to give a better stratification for patients with different prognosis.

## Methods

### Patients

The study enrolled 357 GAC patients from Zhongshan Hospital of Fudan University (Shanghai, China). All the patients included were neoadjuvant therapy naïve and received the semi-elective gastrectomy performed by the same surgical team in 2008 after sufficient preoperative evaluation. All the patients received a radical (R0) resection with a D2 lymphadenectomy. We retrospectively collected the clinical pathological and baseline demographic characteristics of the patients, including age, gender, tumor size, tumor differentiation, Lauren’s classification and tumor stage. We were grateful to the two independent gastroenterology pathologists from Department of Pathology, Zhongshan Hospital for their reassessment for tumor stage according to the 7th Edition of the UICC/AJCC TNM Staging System. Patients with adjuvant chemotherapy received at least one cycle of 5-fluorouracil based chemotherapy. All the patients were followed up until April 2014 with a median follow-up time of 41 months. Overall survival was defined as the time from the date of surgery to the date of death or last visit. All methods were approved by the research medical ethics committee of Zhongshan Hospital, Fudan University and were carried out in accordance with the approved guidelines. Written informed consent on the use of clinical specimens from each patient was achieved.

### Tissue microarray, Immunohistochemical Staining and Evaluation

The immunohistochemical protocols were as previously described^27^, and the tissue microarray was established with formalin-fixed paraffin-embedded surgical specimens. Anti-Indoleamine 2,3-dioxygenase antibody (0.5 mg/ml, ab55305, Abcam, Cambridge, MA, USA) was used as the primary antibody in the immunohistochemical analysis. The density of positive staining was measured by the computerized image system composed of an Olympus CCD camera connected to a Nikon eclipse Ti-s microscope. The stained sections were scanned at ×200 magnification and three independent microscopic fields with the strongest staining were captured by NIS-Element F3.2 software to ensure representativeness and homogeneity. Each photo used an identical setting.

The density of immunostaining was measured by Image-Pro Plus version 6.0 software (Media Cybernetics Inc., Bethesda, MD, USA).Integrated optical density (IOD) of all the positive staining in the sections was measured to give a quantitative assessment for the staining. The mean IOD of the three captured microscopic fields was regarded as the density of IDO expression in the represented tissue. Two observers who were blinded to the patient outcomes and clinical pathological characteristics gave the evaluation of immunostaining. The cut-off points for the definition of high/low expression subgroups were determined by X-tile software^28^.

### Statistical Analysis

SPSS 19.0 (SPSS Inc., IL, Chicago) and R software version 3.0.2 with the “rms” package (R Foundation for Statistical Computing, Vienna, Austria) were used to perform the analyses. Pearson χ^2^ test or Fisher’s exact test was used to compare categorical variables. Continuous variables were analyzed by Student’s t test. Kaplan-Meier analysis was used to determine the overall survival. Log-rank test was used to compare the overall survival of the patients between subgroups. Cox proportional hazards model was used to perform multivariate analysis. Nomogram was generated by R software with “rms” package. Calibration plots for 3- and 5-year overall survival were constructed to examine the performance characteristics of the generated nomogram. The prognostic accuracy was measured by calculating the Harrell’s concordance indices (c-indices). All statistical analyses were two-sided and *p* < 0.05 was regarded as statistically significant.

## Additional Information

**How to cite this article**: Liu, H. *et al.* Increased expression of IDO associates with poor postoperative clinical outcome of patients with gastric adenocarcinoma. *Sci. Rep.* **6**, 21319; doi: 10.1038/srep21319 (2016).

## Figures and Tables

**Table 1:** Associations between IDO expression and clinical pathological characteristics in patients with GAC (n = 357).

Characteristics	All Patients	IDO Expression		
		Low	High	p*
Age (years)				0.880
Mean ± SD†	60.3 ± 11.7	60.2 ± 12.1	60.3 ± 11.3	
Gender				0.054
Male	246	129	117	
Female	111	46	65	
Tumor Size (cm)				0.816
Mean ± SD	3.83 ± 2.27	3.80 ± 2.26	3.86 ± 2.28	
Histology‡				0.927
Differentiated	89	44	45	
Undifferentiated	268	131	137	
Lauren’s Classification				0.241
Intestinal	223	115	108	
Diffuse	134	60	74	
Depth of Invasion				**0.045**
T1	59	35	24	
T2	50	22	28	
T3	62	37	25	
T4	186	81	105	
Lymph Node Metastasis				**<0.001**
N0	126	74	52	
N1	38	21	17	
N2	69	39	30	
N3	124	41	83	
pTNM Stage				0.062
I	80	45	35	
II	79	44	35	
III	198	86	112	
Adjuvant chemotherapy§				0.766
Yes	217	105	112	
No	140	70	70	

^*^χ^2^ test or Student’s t test was performed. ***p*** < 0.05 was regarded as statistically significant.

^†^SD: standard deviation.

^‡^Differentiated: tub1, tub2, pap; Undifferentiated: por1, por2, sig, muc.

^§^Patients with adjuvant chemotherapy received at least one cycle of 5-fluoruracil based chemotherapy.

**Table 2:** Hazard ratios of high IDO expression according to different pathological characteristics.

Characteristics	HR (95% CI)	
	IDO Expression (High vs Low)	p*
Depth of Invasion		
T1 (n = 59)	2.247 (0.634–7.966)	0.210
T2 (n = 50)	1.396 (0.456–4.271)	0.559
T3 (n = 62)	**2.835 (1.459–5.509)**	0.002
T4 (n = 186)	**1.730 (1.184–2.526)**	0.005
Lymph Node Metastasis		
N0 (n = 126)	1.476 (0.738–2.953)	0.271
N1 (n = 38)	1.785 (0.617–5.162)	0.285
N2 (n = 69)	1.568 (0.800–3.074)	0.190
N3 (n = 124)	**1.624 (1.045–2.526)**	0.031
pTNM Stage		
I (n = 80)	1.518 (0.510–4.518)	0.453
II (n = 79)	1.429 (0.651–3.136)	0.373
III (n = 198)	**1.984 (1.397–2.817)**	<0.001
Lauren’s Classification		
Intestinal (n = 223)	**2.509 (1.667–3.775)**	<0.001
Diffuse (n = 134)	1.258 (0.794–1.993)	0.328
Histology‡		
Differentiated (n = 89)	**3.195 (1.612–6.331)**	0.001
Undifferentiated (n = 268)	**1.996 (1.477–2.699)**	<0.001
Adjuvant chemotherapy§		
Yes	**1.700 (1.167–2.477)**	0.006
No	**2.356 (1.398–3.969)**	0.001

^*^Cox proportional hazards model was used. ***p*** <0.05 was regarded as statistically significant.

^‡^Differentiated: tub1, tub2, pap; Undifferentiated: por1, por2, sig, muc.

^§^Patients with adjuvant chemotherapy received at least one cycle of 5-fluoruracil based chemotherapy.
